# Parental and Grandparental Ages in the Autistic Spectrum Disorders: A Birth Cohort Study

**DOI:** 10.1371/journal.pone.0009939

**Published:** 2010-04-01

**Authors:** Jean Golding, Colin Steer, Marcus Pembrey

**Affiliations:** Centre for Child and Adolescent Health, Department of Community Based Medicine, University of Bristol, Bristol, United Kingdom; University of Louisville, United States of America

## Abstract

**Background:**

A number of studies have assessed ages of parents of children with autistic spectrum disorders (ASD), and reported both maternal and paternal age effects. Here we assess relationships with grandparental ages.

**Methods and Findings:**

We compared the parental and grandparental ages of children in the population-based Avon Longitudinal Study of Parents and Children (ALSPAC), according to their scores in regard to 4 autistic trait measures and whether they had been given a diagnosis of ASD. Mean maternal and paternal ages of ASD cases were raised, but this appears to be secondary to a maternal grandmother age effect (P = 0.006): OR = 1.66[95%CI 1.16, 2.37] for each 10-year increase in the grandmother's age at the birth of the mother. Trait measures also revealed an association between the maternal grandmother's age and the major autistic trait–the Coherence Scale (regression coefficient b = 0.142, [95%CI = 0.057, 0.228]P = 0.001). After allowing for confounders the effect size increased to b = 0.217[95%CI 0.125, 0.308](P<0.001) for each 10 year increase in age.

**Conclusions:**

Although the relationship between maternal grandmother's age and ASD and a major autistic trait was unexpected, there is some biological plausibility, for the maternal side at least, given that the timing of female meiosis I permits direct effects on the grandchild's genome during the grandmother's pregnancy. An alternative explanation is the meiotic mismatch methylation (3 M) hypothesis, presented here for the first time. Nevertheless the findings should be treated as hypothesis generating pending corroborative results from other studies.

## Introduction

There is evidence from many parts of the developed world that the prevalence of diagnosed autistic spectrum disorder (ASD) has been rising dramatically. Although evidence from twin studies suggests a strong level of heritability, it is clear that there must also be other factors at play. Parental ages have received some attention - there have been a few case control studies comparing ages of mothers of children with autistic spectrum disorder (ASD) with controls and showing that ASD mothers tended to be older than controls, but these studies either had selected non-population based cases, or had inadequate controls or numbers too small for adequate conclusions. In recent years, however, there have been a number of large population based studies of cases of ASD compared either with all births born over the same period or with a set of controls randomly selected from the population at risk. Their conclusions have varied. For example, there have been three studies from Scandinavia, taking advantage of their birth registries and their facility to link these with case registries. That from Sweden, compared 408 cases with 2040 controls and reported no association with advanced maternal age [1]; the two studies from Denmark covering births from 1984–98 [2] and 1973–98 [3] overlapped considerably, yet they come to different conclusions in regard to parental age. One [2] states that maternal age was significantly associated with autism but that this was secondary to a paternal age effect, whereas the other reports that neither were significant on adjusting for one another [3]. The latter study fell into the trap of failing to take account of collinearity - thus if you have two factors closely correlated such as the ages of each parent, then taking account of both simultaneously will automatically result in neither showing an association with the outcome under consideration. In Western Australia, this problem was circumvented by using a step-wise procedure and offering both maternal and paternal ages [4]. Although both factors were univariably highly associated with ASD (P<0.001), it was only maternal age that entered the equation with an almost 3-fold increase in risk to children of mothers aged 35+ compared with those <25. Conversely a study of Israeli conscripts found increased paternal but not maternal ages [5], but two major studies of births in the USA in 1994 showed independent relationships with both maternal and paternal ages [6], [7].

Thus there is a lack of clarity as to whether it is the age of the mother, or of the father, or both that are related to ASD. In reviewing the literature two publications [8], [9] concluded that both older maternal and older paternal ages played a role. Because of the lack of agreement, we decided to address the topic more broadly. We take advantage of a population based study to assess the parental ages at the birth of the study children with ASD, compared with those of the rest of the population, and also assess whether ages of the preceding generation may be important. We consider not only the children who have been diagnosed, but also the various traits that contribute to the autistic spectrum disorders.

## Materials and Methods

The Avon Longitudinal Study of Parents and Children (ALSPAC) started in September 1990 and aimed to enrol all pregnant women resident in the geographic area of Avon, in south-west England, who had an expected date of delivery in the period April 1^st^ 1991 to December 31^st^ 1992 inclusive. The aim of the study was to assess the contribution of the environment (broadly defined to include both the physical and psychosocial influences) on the health, development and wellbeing of children from the earliest ages [10]. The study also aims to look at the way in which genetic variation influences a variety of outcomes and how these influences may be modified by the environment. In all, 14075 children were born to 13881 mothers, an estimated 80% of the eligible population. A total of 13971 children survived to age 7 years. Ethical approval for the study was obtained from the ALSPAC Law and Ethics Committee and the Local Research Ethics Committees.

A dual approach was made to identify the children from the cohort with ASD. Both the health service records, where a multidisciplinary team had reached this diagnosis, and the education system, where ASD had been given as a reason for special educational needs, were used [11]. A total of 86 children were identified in this way.

In parallel we have looked at traits associated with ASD in the ALSPAC study, and shown that 4 traits are particularly predictive of ASD [12]: the coherence scale of the Children's Communication Checklist (CCC) at about 9 y [13]; the Social and Communication Disorders Checklist (SCDC) at about 8 y [14]; the sociability score from the EAS temperament scale at 38 m [15], and a scale of repetitive behaviour at 69 m derived for the ALSPAC Study. For each scale the higher the results, the more autistic the behaviour. All four traits were highly associated with the ASD diagnosis explaining individually between 10% (sociability) and 46% (coherence) of the log-likelihood.

Here we look at the ages of the parents and the grandparents at the birth of the study child and study parents, respectively, comparing children with and without ASD and the worst 10% of scores for the autistic traits. We used stepwise logistic regression for these dichotomous outcomes. We also analysed the traits as continuous variables using multiple regression.

## Results

### Unadjusted associations with autistic measures

**Figure 1:**
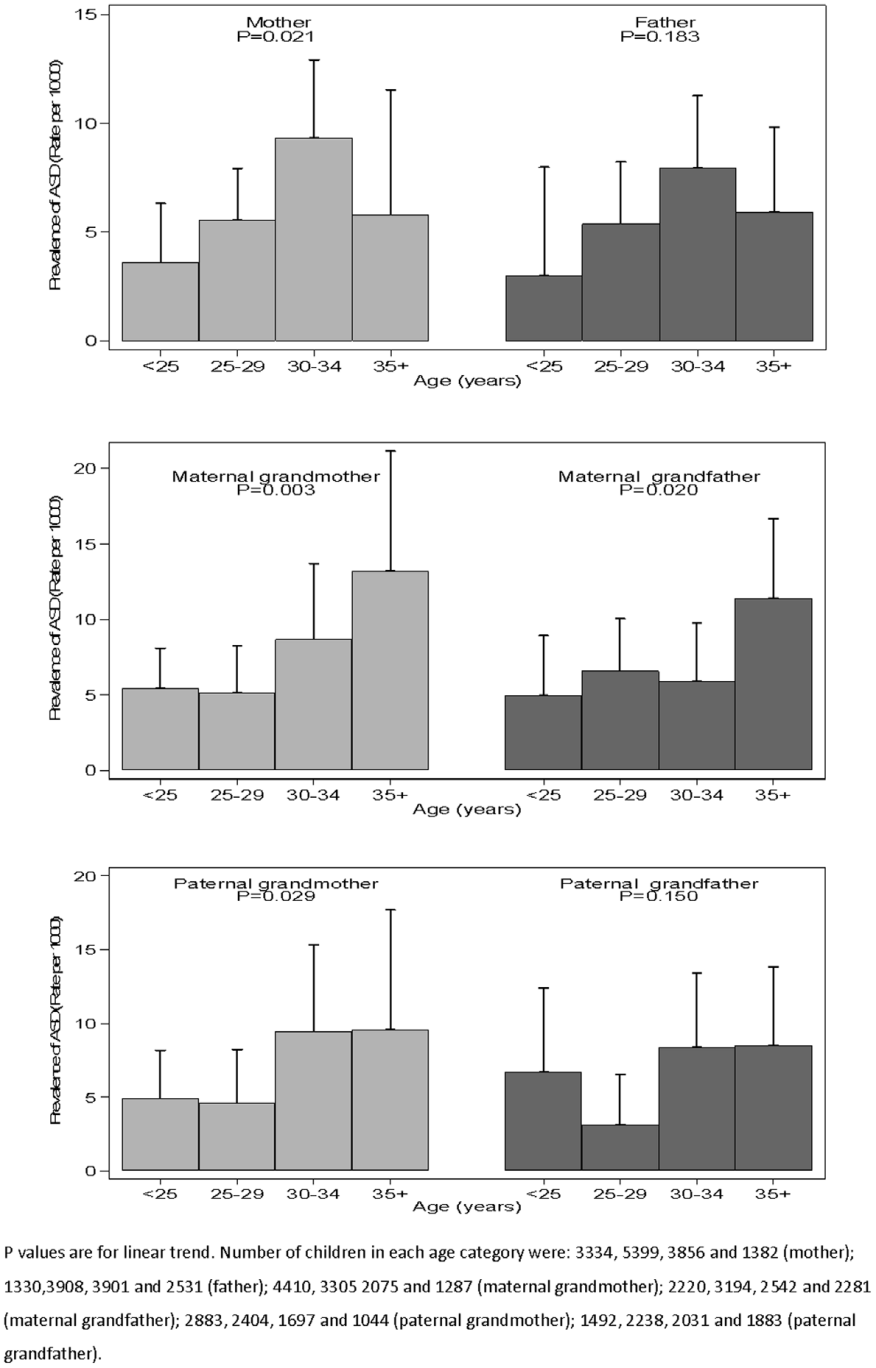
Rates per 1000 of a child having ASD are shown according to ages of parents at child's birth and of grandparents at parents' birth with 95% CIs.

Figure 1 demonstrates the variation in the rate of ASD according to the maternal and paternal ages and shows that there is a lower prevalence of children with an ASD if the parents are young (<25), and increased rates at ages 30–34. The rates of ASD when a parent is 35 or more are similar to the rates at ages 25–29 in this study.

However for the maternal grandparents the rates of ASD in their grandchildren are highest if they were aged 35 or more at the time of birth of the study mother (Figure 1). There is also evidence among the paternal grandparents of an increase in risk when the parents were aged 30 or more.

Comparisons of the mean ages show similar patterns–the mean ages of the parents and grandparents of the children with ASD were higher than found for those without ASD (Table 1). The greatest differences were demonstrated for the grandmothers, with mean differences of 1.90 and 1.97 years for the maternal and paternal grandmothers respectively. Linear regression demonstrates the increase in risk of ASD with each 10 year increase in age, and shows that similar effect sizes are found for ages of mothers and grandmothers (Table 2).

**Table 1:** Comparison of mean ages of parents^a^ and grandparents^b^ of children with and without ASD.

	ASD		Non- ASD		Mean difference
Age of	n	Mean (SD)	n	Mean (SD)	[95% CI]
Mother	86	29.24 (4.97)	13885	27.99 (4.96)	1.26 [0.20, 2.31]^c^
Father	71	31.55 (5.30)	11599	30.71 (5.74)	0.84 [−0.50, 2.17]
Maternal grandmother	76	28.68 (6.20)	11001	26.79 (5.85)	1.90 [0.58, 3.22]^d^
Maternal grandfather	73	31.04 (6.01)	10164	29.88 (6.72)	1.16 [−0.39, 2.71]
Paternal grandmother	51	29.27 (6.43)	7977	27.30 (5.95)	1.97 [0.33, 3.61]^e^
Paternal grandfather	50	31.86 (7.09)	7594	30.40 (6.84)	1.46 [−0.44, 3.37]

a age of parent at birth of study child.

b age of grandparent at birth of the parent.

c P = 0.019.

d P = 0.005.

e P = 0.018.

**Table 2:** Increase in risk [95% confidence interval] of child having ASD for each 10 year increase in age of parents and grandparents adjusted for gender.

Age of	OR [95% CI]	P
Mother	1.60 [1.05, 2.44]	0.029
Father	1.24 [0.84, 1.82]	0.278
Maternal grandmother	1.66 [1.16, 2.37]	0.006
Maternal grandfather	1.27 [0.92, 1.75]	0.140
Paternal grandmother	1.65 [1.07, 2.54]	0.023
Paternal grandfather	1.29 [0.89, 1.87]	0.176

In Tables 3a and b, relationships with the ages are shown for the 4 autistic traits considered. Maternal age was associated with the SCDC trait, such that the younger the mother the more autistic the trait. Coherence showed a similar but less marked younger mother pattern. There were no significant associations with paternal age. There were, however, significant trends for older maternal grandmothers to be associated with worse levels on the Coherence Scale whether analysed according to the worst decile of the scale (P = 0.025, Table 3), or using the continuous scale (P = 0.007, Table 4). There were no other traits showing statistically significant associations with grandparental age.

**Table 3:** Increase in risk [95% confidence interval] of child being in the worst decile of each autistic trait for each 10 year increase in age of parents and grandparents adjusted for gender.

	UNADJUSTED OR [95% CI]			
Age of	Coherence	SCDC^a^	RB^b^	Sociability
Mother^c^	0.92 [0.79, 1.09] (P = 0.337)	0.80 [0.68, 0.93] (P = 0.005)	0.94 [0.79, 1.13] (P = 0.520)	0.96 [0.84, 1.09] (P = 0.529)
Father^c^	1.13 [0.98, 1.29] (P = 0.088)	0.91 [0.79, 1.05] (P = 0.197)	0.95 [0.82, 1.11] (P = 0.557)	0.99 [0.89, 1.12] (P = 0.928)
Maternal grandmother^d^	1.17 [1.02, 1.34] (P = 0.025)	0.99 [0.86, 1.13] (P = 0.839)	0.99 [0.85, 1.16] (P = 0.921)	0.95 [0.84, 1.06] (P = 0.341)
Maternal grandfather^d^	1.13 [1.00, 1.28] (P = 0.052)	0.97 [0.86, 1.10] (P = 0.663)	1.03 [0.89, 1.19] (P = 0.684)	1.00 [0.90, 1.11] (P = 0.936)
Paternal grandmother^d^	1.00 [0.86, 1.17] (P = 0.980)	1.15 [0.98, 1.34] (P = 0.084)	1.04 [0.87, 1.24] (P = 0.691)	1.11 [0.97, 1.26] (P = 0.125)
Paternal grandfather^d^	0.98 [0.85, 1.12] (P = 0.751)	1.07 [0.94, 1.23] (P = 0.316)	1.09 [0.94, 1.28] (P = 0.245)	1.07 [0.95, 1.20] (P = 0.273)

a Social and Communication Disorders Checklist.

b Repetitive behaviour score.

c age at birth of study child.

d age of grandparent at birth of the parent.

**Table 4:** Linear regression of parental and grandparental ages on autistic traits; results per 10 years increase in age, high scores indicate more autistic like traits adjusted for gender.

	UNADJUSTED REGRESSION COEFFICIENT [95% CI]			
Age of	Coherence	SCDC^a^	RB^b^	Sociability
Mother^c^	−0.13 [−0.23, −0.03] (P = 0.009)	−0.30 [−0.48, −0.13] (P = 0.001)	−0.01 [−0.03, +0.01] (P = 0.509)	+0.03 [−0.10. +0.16] (P = 0.676)
Father^c^	+0.02 [−0.06, +0.11] (P = 0.594)	−0.12 [−0.27, +0.03] (P = 0.110)	+0.00 [−0.02, +0.02] (P = 0.794)	−0.02 [−0.13, +0.10] (P = 0.779)
Maternal grandmother^d^	+0.12 [+0.03, +0.20] (P = 0.007)	−0.11 [−0.25, +0.04] (P = 0.152)	+0.00 [−0.01, +0.02] (P = 0.645)	−0.01 [−0.13, +0.10] (P = 0.799)
Maternal grandfather^d^	+0.07 [−0.01, +0.14] (P = 0.084)	−0.08 [−0.21, +0.05] (P = 0.233)	+0.01 [−0.01, +0.02] (P = 0.410)	+0.00 [−0.10, +0.10] (P = 0.994)
Paternal grandmother^d^	−0.01 [−0.10, +0.09] (P = 0.914)	+0.09 [−0.08, +0.25] (P = 0.301)	+0.00 [−0.02, +0.02] (P = 0.919)	+0.09 [−0.03, +0.22] (P = 0.150)
Paternal grandfather^d^	−0.05 [−0.14, +0.03] (P = 0.209)	+0.03 [−0.12, +0.18] (P = 0.684)	+0.01 [−0.01, +0.03] (P = 0.290)	+0.04 [−0.07, +0.15] (P = 0.469)

a Social and Communication Disorders Checklist.

b Repetitive behaviour score.

c age at birth of study child.

d age of grandparent at birth of the parent.

### Correlations between ages

There is, however, a strong correlation between ages of spouses. Table 5 shows that there is also a strong correlation between the parent's age at the birth of the study child and the age of each grandparent at the birth of the study parent. Although the correlations between the ages of the maternal and the paternal grandparents are low (0.05–0.08), those between the parents and grandparents are higher but still modest (r = 0.17 to 0.26). The correlation between the ages of spouses is higher for grandparents than for parents.

**Table 5:** Correlations between parental and grandparental ages–all study families.

Age of	M	F	MGM	MGF	PGM	PGF
Mother (M)	1.000					
Father (F)	0.661	1.000				
Maternal grandmother (MGM)	0.255	0.195	1.000			
Maternal grandfather (MGF)	0.230	0.205	0.809	1.000		
Paternal grandmother (PGM)	0.190	0.189	0.076	0.058	1.000	
Paternal grandfather (PGF)	0.172	0.177	0.068	0.050	0.790	1.000

[ages are those of parents at the birth of the study child, and of grandparents at the birth of the study parent].

All correlations were statistically significant p<0.001.

### Adjusted associations

#### ASD

To assess which of the highly correlated ages were most important in regard to ASD, forward step-wise multiple regression was used; on offering all the 6 age variables only one entered the model–the maternal grandmother's age: OR 1.66, [95% CI 1.16, 2.37] per 10 year increase in age (P = 0.006). In the presence of this factor the mother's age showed an odds ratio (OR) of 1.39 (95% CI 0.86, 2.24; P = 0.177). The paternal grandmother's age in the presence of the maternal grandmother's age, however, did exhibit an effect that bordered on statistical significance (OR 1.54, 95%CI 0.97, 2.45; P = 0.069).

In order to ensure that the analysis using the ages as continuous variables were not hiding important non-linear effects, we used the ages as 4 categorical variables as shown in Figure 1, and used stepwise logistic regression to determine which factors would enter. Again it was only the maternal grandmother's age that entered the model (P = 0.004); once again, however, the paternal grandmother's age was of borderline significance in the presence of the maternal grandmother's age (P = 0.066) and the mother's age given that of her own mother was not statistically significant (P = 0.169). Numbers were too small for detailed multivariable analysis, but the only unadjusted socio-demographic or psychosocial factor that was significantly associated with ASD was paternal social class. Taking this into account the maternal grandmother effect size did not change substantially (OR = 1.69, 95%CI 1.15, 2.49, P = 0.007); n = 9957.

Analyses were also repeated taking into account the non-independence of multiple births. Both maternal and paternal grandmother effects were strengthened and exerted independent effects–OR = 2.04 [1.26, 3.29] and 1.06 [1.01, 1.11] respectively. But in all other respects, the conclusions were not affected by these adjustments.

#### Autistic traits

In regard to the coherence trait, only the age of the maternal grandmother entered the logistic regression, indicating that the child was 1.17 times more likely to be in the worst decile of this trait for each 10 year increase in grandmother's age [95% CI 1.02, 1.34], P = 0.025. When the scale was used linearly, both the grandmother's and the mother's ages entered–the grandmother showing an adverse effect with increasing age (b = 0.14, 95%CI 0.06, 0.23, P = 0.001), whereas maternal age independently showed the reverse association (b = −0.14, 95% CI −0.25, −0.03, P = 0.011). The only other trait that showed any independent association with an age measure was maternal age which entered the SCDC model with OR = 0.80 [95% CI 0.68, 0.93] for the worse decile and b = −0.30 [95%CI −0.48, −0.13] for linear trend (P = 0.005 and 0.001 respectively): i.e. the SCDC trait score increased as the mother's age decreased.

Analysis to assess whether the relationship of the maternal grandmother's age with the ‘Coherence’ score was an artefact first investigated over 100 environmental, behavioural or psychological measures that might be related to this trait. On assessment of the unadjusted associations we selected 35 which were highly significant (P<0.001). Each of these were then examined to determine their effect on the regression coefficient for the ages. Only maternal personality at 18 w gestation (−9.8%) and parity (−2.1%) attenuated the age effect. In all, 7 variables had an effect between 10 and 20% (maternal social class, paternal education, paternal grandmother's education, repetitive behaviour at 30 m, maternal depression scale at 32 weeks gestation and 8 months postnatally and a Family Adversity Scale covering the first 2 years of the study child's life). There remained 6 factors with an effect >20%. (maternal education level, housing tenure, child's passive smoke exposure at 15 m, whether the TV was on in the afternoons at 18 m, and the Family Adversity Level in pregnancy and in the 3^rd^–4^th^ year of the child's life). Maternal age was also kept in the analysis. The regression coefficients for maternal age and for maternal grandmother's ages on inclusion of the 6 factors were b = −0.046 [95% CI −0.173, + 0.080] (P = 0.474) and b = 0.217, [95% CI 0.125, 0.308] (P<0.001) respectively per 10 year increase in age (n = 5989). Further analysis taking account of the child's IQ resulted in b = 0.207 [95% 0.115, 0.299] (P<0.005) per 10 year increase in maternal grandmother's age (n = 4770).

Adjustments for multiple births did not alter the conclusions.

#### Missing data

As Table 1 shows, varying amounts of missing data existed for different ages varying from zero for maternal age (data obtained from registration details) to 45% for paternal grandfather age (data obtained from a partner questionnaire administered during pregnancy). Complete data were available for 5851 (42%) children. Analyses using this sample suggested a similar dominant effect for maternal grandmother age as reported in Table 2 and in *Adjusted associations* above. Athough this age effect was somewhat higher for the restricted sample OR = 2.17 [95% 1.32, 3.55], this effect was not statistically different from the result of OR = 1.65 obtained for 11075 children (interaction p = 0.127). This result may imply the transition from observed to the total sample of 13971 children will also have a minimal impact on the associations reported.

#### Confounding

The associations of outcomes and predictors with socio-demographic variables are shown in Table 6. While strong associations existed, in general, there was little evidence of confounding. For instance, with gender, associations were only present for outcomes but not for predictors. For others, such as social class, education and housing, the direction of associations between outcomes and predictors tended to occur in opposite directions. This may explain the result above whereby adjustment tended to strengthen the associations.

**Table 6:** Association of ASD, traits and age variables with socio-demographic variables.

	Gender	Birth order	Social class	Education	Housing
ASD	0.152 (1.403)***	1.525 (1.280)	0.803 (1.104)*	0.984 (1.094)	0.898 (1.196)
**Traits**					
Coherence	−0.461 (0.046)***	0.164 (0.048)***	0.055 (0.020)**	−0.129 (0.020)***	0.122 (0.039)**
SCDC	−0.856 (0.082)***	−0.161 (0.084)	0.089 (0.034)**	−0.119 (0.034)***	0.287 (0.067)***
RB	−0.055 (0.010)***	0.002 (0.010)	−0.000 (0.004)	−0.006 (0.004)	0.033 (0.008)***
Sociability	−0.331 (0.062)***	0.688 (0.063)***	0.070 (0.026)**	−0.191 (0.025)***	−0.011 (0.049)
**Ages**					
M	−0.155 (0.087)	2.656 (0.086)***	−1.061 (0.034)***	1.050 (0.033)***	−2.068 (0.061)***
F	−0.245 (0.108)*	2.409 (0.108)***	−0.954 (0.043)***	0.877 (0.043)***	−1.689 (0.081)***
MGM	−0.078 (0.113)	0.201 (0.116)	−0.503 (0.047)***	0.685 (0.045)***	−0.888 (0.086)***
MGF	−0.010 (0.135)	0.271 (0.139)	−0.529 (0.056)***	0.744 (0.054)***	−0.729 (0.105)***
PGM	−0.165 (0.136)	0.453 (0.137)***	−0.536 (0.056)***	0.553 (0.055)***	−0.796 (0.105)***
PGF	−0.357 (0.159)*	0.545 (0.161)***	−0.524 (0.066)***	0.562 (0.065)***	−0.782 (0.125)***

Gender effect is for females. Birth order coded as 0 (no older siblings) and 1 (one or more older siblings). Paternal social class coded as I, II, III non-manual, III manual and IV+V combined. Maternal educational qualifications coded as none or CSE, vocational, O level, A level or degree. Housing coded as mortgaged/owned, local authority housing and other. Traits are coded so that low scores reflect a more favourable response. Traits are labelled as in Table 3. Age variables are labelled as given in Table 4. Reported effect sizes relate to a linear trend. Effect sizes are ORs for ASD and regression coefficients for other outcomes. Standard errors (or exp(SE) for ASD) are given in parentheses.

## Discussion

There have been a number of population studies showing that children with ASD are more likely to be born to older parents. In this study, for the first time to our knowledge, we have assessed possible relationships with the ages of the parents' own parents at the time of their birth. We had no prior hypotheses as to whether we would find relationships through the male or female line, but found that the ages of the grandmothers were higher than expected, and that the relationship with the maternal grandmother was statistically significant (P = 0.006).

There is increasing recognition that trying to find a biological basis for syndromes such as ASD is probably best served by study of the component traits [16], [17]. Elsewhere we have investigated within ALSPAC the associations between ASD and 90 different trait scales. Of these, 4 were identified as independently associated with ASD, thus providing a compromise between parsimony and explanatory power [12]; these were coherence, social communication, sociability and repetitive behaviour; together they accounted for 54% of the variance. Of the 4 traits the coherence scale showed the strongest relationship with ASD. Therefore in this study, as well as looking at study subjects which have an ASD diagnosis, we have assessed relationships with these 4 traits. We showed that the coherence trait, whether treated as a continuous scale or dichotomously studying the worst decile of the distribution, was significantly associated with the maternal grandmother's age.

In regard to whether the grandmother's age effects might be explained by older women being more likely to have daughters (i.e. study mothers) with autistic traits, we examined the information that had been collected from the study parents during pregnancy and later. For ASD there was no hint of any associations with maternal history of child guidance or speech therapy or of current unusual personality traits (data not shown). The parents had similar social networks and education levels to the rest of the population, but there were positive associations with paternal social class such that the children of fathers in non-manual as opposed to manual occupations were at increased risk of ASD [11]. Taking this into account the maternal grandmother effect remained strongly associated.

Although analyses of data on the ASD cases suffer from lack of statistical power, this was not true of the trait measures. The Coherence trait which has been shown to be closely related to a diagnosis of ASD in our data, had sufficient power for a number of factors to be taken into account. Unadjusted analyses concerning over 100 potential confounders were examined and 6 showing a change of at least 20% in the effect size were selected for multivariable analysis. The result was an increase in the regression coefficient from 0.142 [95% CI 0.057, 0.228] to 0.217 [95% CI 0.125, 0.308] per 10 year increase in maternal grandmother's age. In a further analysis we took the child's IQ into account in order to ensure that it was the Coherence trait we were assessing rather than some effect of the child's intellectual ability, but again this made little difference to the relationship with maternal grandmother's age.

**Figure 2:**
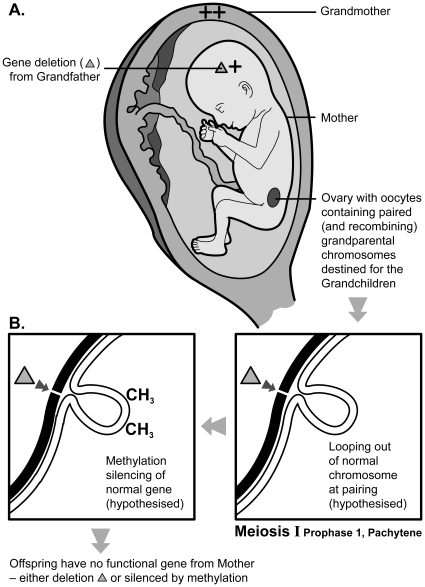
Three generations of genotypes are illustrated (A): that involving the grandmother's pregnancy, her female fetus and the fetal ovary that contains the emerging genotype of the grandchild. The grandmother has two normal, wild type genes (++). The fetus has a deletion of the gene inherited from grandfather (**▵**) which confers some susceptibility to autistic spectrum disorder. The hypothesised mispairing of the grandparental chromosomes at the site of the gene deletion (**▵**) in the fetal oocytes is shown (B). The chromosome containing the wild type gene loops out at meiotic pairing and this gene becomes liable to be silenced by DNA methylation. This results in no grandchild receiving a functional gene.

This maternal grandmother's age effect, found for both ASD and for one of the major autistic traits, was unexpected and will need replication, but it is biologically plausible because of the timing of meiosis in females. As Figure 2 illustrates, the paired (and recombining) grandparental chromosomes that will be transmitted from the mother to her offspring are already there in the fetal ovary from the second trimester of the grandmother's pregnancy. This permits a direct grand-maternal effect on the germ line that will be passed to the grandchild.

Although in this study maternal and paternal ages were raised, our analysis indicates that the primary association with ASD risk is the maternal grandmother's age. If this is confirmed in other studies, it suggests three broad possibilities–

- Women with older mothers are more likely to push for a diagnosis for their child. Although there is no literature on this it is known that older mothers tend to recognise signs of ASD earlier [18], and it is likely that older grandmothers would do the same. However this does not explain the association between maternal grandmother's age and the Coherence trait, since the scale involved was completed by a large population of mothers, and did not depend on any diagnosis.
- There is something about early stages of meiosis I in the fetal ovaries that is particularly sensitive to maternal age effects, with the (genomic) malfunction being played out in the grandchild.
- An inherited risk factor is amplified in some way by passage across at least one generation, i.e. the maternal age effect increases the ASD risk in the daughter to a lesser degree than in the grandchild. This could happen in two ways: enrichment for those germ cells that happen to carry an increased ASD risk or some progressive change to the genome. Candidates for the former include an age-related loss of selection against oogonia or oocytes with *de novo* genetic damage or indeed a proliferative advantage of cells with an ASD risk genotype. Candidates for progressive change to the genome include a dynamic triplet repeat mutation, as in fragile X, but where older maternal age is associated with *de novo* premutations, or some epigenetic spreading of the genetic malfunction during transmission to the next generation, e.g. at meiosis.

The last two scenarios imply a transmitted change in the genome or other heritable material. Twin [19] and sibling risk studies [2] show that ASD is highly heritable with monozygotic twins having 92% concordance compared to 10% in dizygotic twins. It is usually assumed that this heritability is genetic, although transmission of molecular information other than through DNA sequence (e.g. microRNAs) cannot be ruled out [20]. Recently, specific genetic risk factors for ASD have been reported, but they are very heterogeneous. To date one common single nucleotide polymorphism (SNP) variant associated with ASD risk has been reported [21], but there have been several studies showing an excess of rare microdeletions and in some cases microduplications in ASD compared with controls [22]–[25]. These so-called copy number variants (CNVs) may be inherited from a parent or arise *de novo*, i.e. new mutations. Some studies [24] deliberately excluded inherited CNVs in an attempt to enrich for causal variants, whilst a recent study of smaller deletions and duplications involving the coding regions (exons) of genes demonstrates that possible susceptibility CNVs are often inherited [25]. However when inherited in multiplex families (i.e. with several affected family members) there is imperfect segregation with ASD with affected siblings often *not* inheriting the exonic deletion [25]. This result is less easy to explain in a multiplex family than some asymptomatic family members carrying the exonic deletion, if indeed the inherited deletion is contributing to the familial ASD.

There are few published data on the possible mediators of (grand)maternal age effects that help distinguish between scenarios b and c above. A recent study of reproductive and epigenetic outcomes with aging mouse oocytes found morphological abnormalities (increased trophoblast giant cells) in the resulting placentae [26], which raises the possibility of impaired transplacental transfer of nutrients or metabolic signals to the germ line in the fetal ovaries. Despite early work suggesting a reduction in DNA methyltransferases with age, the authors found no impairment of DNA methylation at imprinted genes and more widely across the genome.

Given the ongoing generation of *de novo* CNVs, deterioration with maternal age in the ‘surveillance’ mechanisms for eliminating cells with genetic imbalance is a possibility. Less selection against oogonia with a high CNV load in the fetal ovaries would increase the ASD risk. Recent work in relation to Down syndrome indicates that ovarian trisomy 21 mosaicism is common and the maternal age effect is likely to be, in part, a change in oocyte selection [27]. DNA fragmentation is increased in oocytes from older mice [28] as is mitochondrial dysfunction [29] which in turn may compromise the cell quality surveillance role of mitochondria through apoptosis [30]. However, human research on the development of oocytes and influences on female meiosis is necessarily limited, so family based genetic and epigenetic studies might prove more productive.

One possibility, prompted by our data suggesting a maternal grandmother age effect in ASD, would be to test what we have called the meiotic mismatch methylation (3 M) hypothesis (Fig. 2b). Mispairing of a chromosome bearing an ASD risk deletion with the normal homologous chromosome during early meiosis I (in the ovaries of the mother as a fetus) might lead to methylation and silencing of the normal gene. If this were the case then *all* the mother's children would inherit a risk allele, either the deletion or a gene silenced by DNA methylation. All other ASD risk factors being equal, such a meiotic mismatch methylation (beginning during the grandmother's pregnancy) would lead to a higher risk of ASD in the grandchildren than in the mother. It is known that meiotic mismatches are accommodated in various ways and the looping out illustrated in Fig. 2b has been known as one such mechanism for a long time [31].

Beyond the inherited ASD risk deletion (or duplication) itself, are there any distinctive features of genomic regions harbouring CNVs that might predispose to meiotic mismatch and DNA methylation? Such genomic regions tend to contain repeated DNA sequences (that are implicated in the generation of the CNV in the first place [32]), and such sequences can lead to other forms of mismatch at meiosis such as non-allelic-homologous-pairing. The key question is whether these perturbations of meiotic pairing trigger spread of DNA methylation to silence the otherwise functional normal gene (wild type allele). DNA methylation is believed to have first evolved as a genome defence system to silence transposons and the like and such sequences are preferentially methylated [33], so the same genomic architecture that predisposes to CNVs might also attract DNA methylation. The 3 M hypothesis is testable in family studies such as reported by Bucan et al [25] where a specific ASD risk CNV is segregating. It would predict that those affected members *not* carrying the CNV would have DNA methylation silencing of the wild type allele.
